# Restoration of natural somatic sensations to the amputees: finding the right combination of neurostimulation methods

**DOI:** 10.3389/fnins.2024.1466684

**Published:** 2024-11-25

**Authors:** Gurgen Soghoyan, Artur R. Biktimirov, Nikita S. Piliugin, Yury Matvienko, Alexander Y. Kaplan, Mikhail Y. Sintsov, Mikhail A. Lebedev

**Affiliations:** ^1^Vladimir Zelman Center for Neurobiology and Brain Rehabilitation, Skolkovo Institute of Science and Technology, Moscow, Russia; ^2^Laboratory of Experimental and Translational Medicine, School of Biomedicine, Far Eastern Federal University, Vladivostok, Russia; ^3^Motorica Research Center, Moscow, Russia; ^4^Faculty of Mechanics and Mathematics, Lomonosov Moscow State University, Moscow, Russia; ^5^I. M. Sechenov Institute of Evolutionary Physiology and Biochemistry, St. Petersburg, Russia

**Keywords:** neuroprosthetics, neuromodulation, sensory restoration, embodiment, peripheral nerve stimulation, sensory feedback, EEG, spinal cord stimulation (SCS)

## Abstract

Limb amputation results in such devastating consequences as loss of motor and sensory functions and phantom limb pain (PLP). Neurostimulation-based approaches have been developed to treat this condition, which provide artificial somatosensory feedback such as peripheral nerve stimulation (PNS), spinal cord stimulation (SCS), and transcutaneous electrical nerve stimulation (TENS). Yet, the effectiveness of different neurostimulation methods has been rarely tested in the same participants. Meanwhile, such tests would help to select the most effective method or a combination of methods and could contribute to the development of multisensory limb prostheses. In this study, two transhumeral amputees were implanted with stimulating electrodes placed in the medial nerve and over the spinal cord epidurally. PNS and SCS were tested in each participant as approaches to enable tactile and proprioceptive sensations and suppress PLP. Both PNS and SCS induced sensation in different parts of the phantom hand, which correlated with cortical responses detected with electroencephalographic (EEG) recordings. The sensations produced by PNS more often felt natural compared to those produced by SCS. Еvoked response potentials (ERPs) were more lateralized and adapted faster for PNS compared to SCS. In the tasks performed with the bionic hand, neurostimulation-induced sensations enabled discrimination of object size. As the participants practiced with neurostimulation, they improved on the object-size discrimination task and their sensations became more natural. А combination of PNS and TENS enabled sensations that utilized both tactile and proprioceptive information. This combination was effective to convey the perception of object softness. In addition to enabling sensations, neurostimulation led to a decrease in PLP.

**Clinical trial registration:**

https://clinicaltrials.gov/, identifier, #NCT05650931.

## 1 Introduction

The prosthetic devices for the amputees could be improved with the technology of brain-computer interfaces (BCIs), the systems that connect to the brain to enhance or restore motor functions (Lebedev and Nicolelis, 2017). In BCIs, intentions to perform voluntary movements are decoded from the activity of different areas of the nervous system, which enables control of external effectors even in such complex tasks as handwriting (Willett et al., 2021). The utilization of BCI technologies has the potential to greatly enhance the quality of life of individuals suffering from severe neurological disorders (Daly and Wolpaw, 2008; Wang et al., 2010). For example, use of electromyographic (EMG) decoders is a common and rather intuitive approach to controlling prostheses by the amputees (Al-Timemy et al., 2013).

Even when using sophisticated prosthetic limbs, users still experience difficulties if a prosthesis does not provide sufficient sensory feedback (Kyberd et al., 2007; Lee Childers et al., 2014; Schieber, 2016). This problem is exacerbated by the presence of phantom limb pain (PLP) experienced by up to 80% of amputees (De Nunzio et al., 2018; Flor, 2002). It has been previously proposed that different neurostimulation methods could be used to suppress PLP such as invasive motor cortex stimulation (Ramos-Fresnedo et al., 2022), transcranial magnetic stimulation (Przeklasa-Muszyńska, 2014) and transcranial direct current stimulation (Bolognini et al., 2015; Przeklasa-Muszyńska, 2014). Additionally, neurostimulation is suggested as a potential approach to simultaneously treat PLP and enable neuroprosthetic feedback (Soghoyan et al., 2023). Thus, peripheral nerve stimulation (PNS; Kumar and Rizvi, 2014) could be used to both implement prosthetic sensations and suppress PLP (Soghoyan et al., 2023). In addition to PNS, spinal cord stimulation (SCS) is applicable to treat PLP and other types of neuropathic pain (Kumar and Rizvi, 2014; Knotkova et al., 2021). PNS and SCS suppress PLP by inhibiting the effects of the pathological discharges generated in the neuromas and affecting the spinal (Foell et al., 2011) and cortical (Zheng et al., 2021) activity. A preferred neurostimulation system for the amputees would be a bidirectional one where stimulation parameters are set based on PLP-related changes in neural activity (Kleeva et al., 2022). In addition to PNS and SCS, such a system could incorporate transcutaneous electrical nerve stimulation (TENS; Flor et al., 2001) and targeted muscle reinnervation (TMR; Mioton et al., 2020).

Neuroprostheses for enabling sensations have been developed for different sensory modalities, including the very successful auditory implants and visual prostheses which are rapidly developing using stimulation of the retina (Ayton et al., 2020), visual nerve (Finn and LoPresti, 2002), thalamus (Panetsos et al., 2011) and cortex (Fernandez, 2018) to generate visual sensations. Progress has been made in the development of sensorized hand prostheses for the amputees, as well (Soghoyan et al., 2023; Bensmaia et al., 2023; Raspopovic et al., 2021; Makin and Bensmaia, 2017). Yet, more research is needed for improving the practicality of these systems. Steps toward the development of the practical somatosensory prostheses have been made, including neurostimulation-based systems for enabling sensations of object size and texture (Raspopovic et al., 2021; Oddo et al., 2016). Here, the embodiment of a prosthesis (Nelson et al., 2020) is considered the ultimate criterion of success and obstacles include instability of neurostimulation-induced sensations (Cuberovic et al., 2019). Given that several neurostimulation approaches exist that are capable of generating somatic sensations, it is reasonable to suggest that an individually adjusted combination of these methods could be particularly effective to enable prosthetic sensations and suppress PLP in amputees.

Here we evaluated the effectiveness of both the PNS and SCS in two transhumeral amputees. The parameter values of these neurostimulation methods were explored to improve the naturalness of sensations felt in the phantom limb. Furthermore, the patients learned to perform several sensory discrimination tasks with a bionic hand, where PNS, SCS, and TENS provided sensory inputs. This approach worked well to simultaneously generate proprioceptive and tactile sensations in the tasks where the bionic hand was used to assess object softness. The measurements of cortical evoked response potentials for different types of neurostimulation further clarified how artificial somatic sensations were processed. Overall, these findings improve our understanding of how neurostimulation could provide near-natural multimodal sensations for the prosthetic control while eliminating PLP.

## 2 Materials and methods

### 2.1 Surgery and patients

Two amputees participated in the study; both suffered from PLP. S12 and S13 are male participants with transhumeral amputation on the left side. At the time of this study, S12 was 41 years old and S13 was 42 years old. Before the injury, both patients worked as manual laborers with the right dominant hand. S12 underwent amputation more than 21 months before the study, while S13 underwent amputation 7 months before. Both patients filled the Pain detect (PD), DN4, Hospital Anxiety and Depression Scale (HADS), SF-36 questionnaires and indicated their level of pain using visual analogue scale (VAS). They also marked phantom-hand locations where the pain was felt. Supplementary materials #1 contains the detailed data on the patients and the results of their pre-surgery examination.

The study was approved by the Ethical Committee of the Far East Federal University (FEFU) Biomedicine school (Protocol #4; April 16, 2021). Each patient signed the informed consent form prior to participating in the experiments. All procedures were in accordance with the Helsinki Declaration. The study is registered as a clinical trial on platform #NCT05650931.^1^

**Figure 1:**
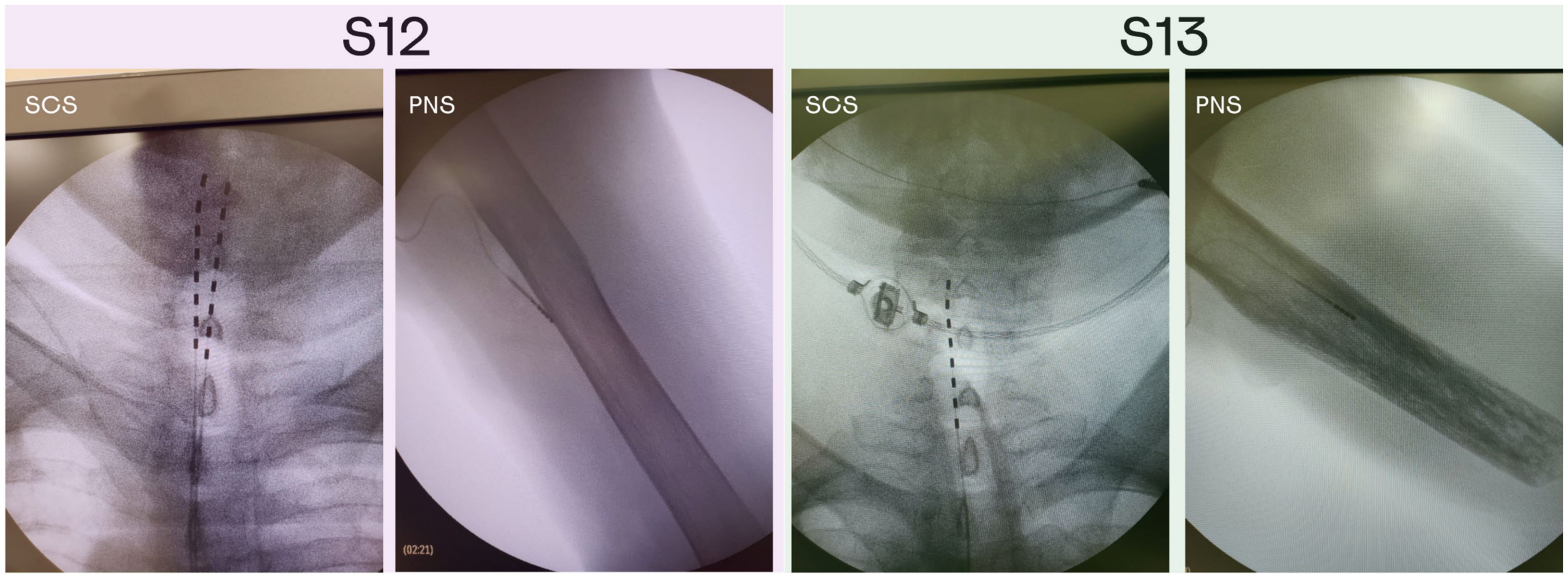
X-ray images of the PNS and SCS electrodes position in patients S12 and S13. Patient S12 was implanted with two electrodes in the area of intumescentia cervicalis of the spinal cord and one electrode in the medial nerve of the left hand. Patient S13 was implanted with two electrodes in the area of intumescentia cervicalis of the spinal cord and one electrode in the medial nerve of the left hand.

Implantation surgeries were performed at the Medical Сenter of FEFU. Eight-contact electrodes (Directional Lead for the St. Jude Medical Infinity™ DBS System; Abbot; United States) were implanted in the median nerve of the left arm in both patients under endotracheal anesthesia; one electrode per patient. To implant the PNS electrodes, the epineurium was disected under a surgical microscope. The electrodes were placed in the space between the nerve fascicles (Planitzer et al., 2014). Additionally, both patients received implants in the area of intumescentia cervicalis of the spinal cord (Figure 1) on the left side. SCS cylindrical electrodes (Vectris Surescan Trail MRI 1 × 8 compact 977D260; Medtronic; United States) were implanted under local anesthesia and X-ray control. For the implantation of the spinal electrodes, a puncture of the posterior epidural space was performed at the level of T6-7 under local anesthesia. Patient S12 was implanted with two SCS leads, and S13 was implanted with one lead. Initially, patient S12 underwent the surgery where one electrode was implanted. However, this SCS electrode migrated to the midline of the spine on the 3rd day after the surgery. To correct this problem, an additional electrode was implanted in the proximity to the first one. This second electrode was used to deliver SCS in this study.

**Figure 2:**
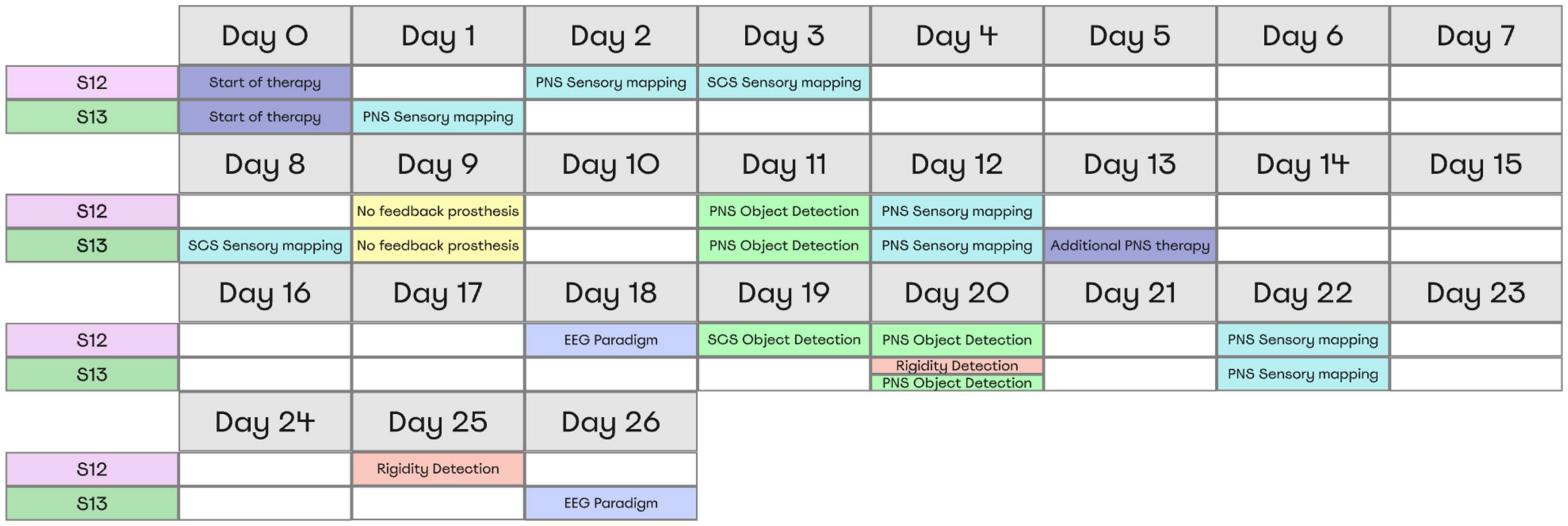
Overview of Experimental Procedures and Timing for Both Subjects. All tests and their respective timing for each patient are shown in a table representing the moments when subjects completed the tasks. Each color represents a separate experimental paradigm.

Over the course of the study, two stimulation paradigms were used: stimulation that continued throughout the day to treat PLP, and experimental stimulation to study different aspects of the prosthetic sensations. The stimulation parameters for PLP treatment (100–1,000 μs pulses at 40–100 Hz) were adjusted individually for each patient, which improved the treatment effect. With these adjustments, patient S12 was treated with PNS over the course of 26 days. Patient S13 received SCS treatment during the first 13 days; then, PNS and SCS programs were combined for the remaining 13 days. The neurostimulation evoked sensations in the phantom-hand area where the patients experienced PLP. They used diaries to daily mark the level of PLP suppression according to a visual analogue scale. Throughout the study, we conducted a series of experimental procedures as outlined in Figure 2. Minor inconsistencies in the schedules for the two subjects were due to medical recommendations and the availability of necessary equipment.

### 2.2 Sensory mapping

#### 2.2.1 PNS

PNS evoked sensations in the phantom hands of both participants. During sensory mapping we switched between different stimulating site pairs and ask patients to describe the sensations that they experience. To measure the changes in the PNS-evoked sensations that occurred over the course of this study, sessions of sensory mapping were conducted in the beginning (day 1 or day 2), in the middle (day 12) and at the end (day 22) of the study. We were particularly interested in the contact sites that evoked sensations in the phantom limb. During the first mapping session, the stimulation frequency was 50 Hz, and during the second and third session it was increased to 100 Hz to improve sensation naturalness. Pulse width was 100 μs for all tests.

For each electrode pair, stimulation amplitude was initially 0 mA, and it was then gradually increased from 0.1-mA steps. For each step, the participants reported sensation intensity on a 0 to 10 scale where 0 corresponded to no sensation and 10 corresponded to an uncomfortably strong sensation. For the intensity equal to 5, the patients filled the PerceptMapper questionnaire (Nanivadekar et al., 2020) where they described the sensation qualitatively and marked their location on an image of the hand displayed on a computer screen. We used the questionnaire form with minor changes and translated it to Russian language which is described in details on our previous study (Soghoyan et al., 2023). Then, another electrode pair was selected and tested. To describe the stimulation-evoked sensations the participants selected the terms from the listed in the questionnaire, and they used sliders to report sensation naturalness. The patients reported two classes of sensations: naturalistic and non-naturalistic (Table 1). The counts of different kinds of reported sensations were quantified as statistical distributions where data was normalized by the number of trials per day. This analysis assessed the effectiveness of PNS for evoking natural sensations.

**Table 1:** Subdivision of stimulation-evoked sensations into non-naturalistic and naturalistic.

**Non-naturalistic**	**Naturalistic**
Electric shock, pulsing, vibration, flutter	Pressure, touch, shock, prick, urge to move, itch

To describe the stimulation-evoked sensations the participants selected the terms from the questionnaire descriptors. Based on patients’ reports, descriptors were split into two classes: naturalistic and non-naturalistic.

To calculate the density of neurostimulation projection sites on the phantom hand, we averaged the hand images where patients had marked their phantom sensations.

#### 2.2.2 SCS

Both patients were implanted with the electrodes for SCS, which allowed us to compare the effects of SCS and PNS. Spinal mapping was arranged the same way as the mapping using PNS: hand images were used for the mapping, the evoked sensation intensity was reported with sliders, and the participants described their sensations using the questionnaire. A single SCS mapping session was conducted in each patient: on day 3 in patient S12 and on day 8 in patient S13, since the first subject had to follow an additional surgery. The access to patients was regulated by the medical specialists. In each of these sessions and in each patient, one electrode with eight stimulating sites was used.

### 2.3 Object size detection

#### 2.3.1 Experimental design

In this task PNS feedback enabled discriminating object size, while prosthetic hand grasped rigid cylinders made of PLA plastic (Video 1). Simultaneously, the signals from the prosthetic sensor of aperture were converted into PNS patterns. The cylinders came in three sizes: small (20 mm in diameter), medium (40 mm), and large (60 mm), that were shown to patients before the experiments. The patients controlled the prosthesis grasping using an external controller with two buttons: “open” and “close.” When a patient initiated the grasping, closing the prosthesis aperture (measured with prosthetic finger encoders) resulted in an increase in the stimulation amplitude (see section Stimulation settings for sensorimotor tests). We describe this approach as artificial proprioception because neurostimulation mimicked sensing of hand configuration.

**Figure 3:**
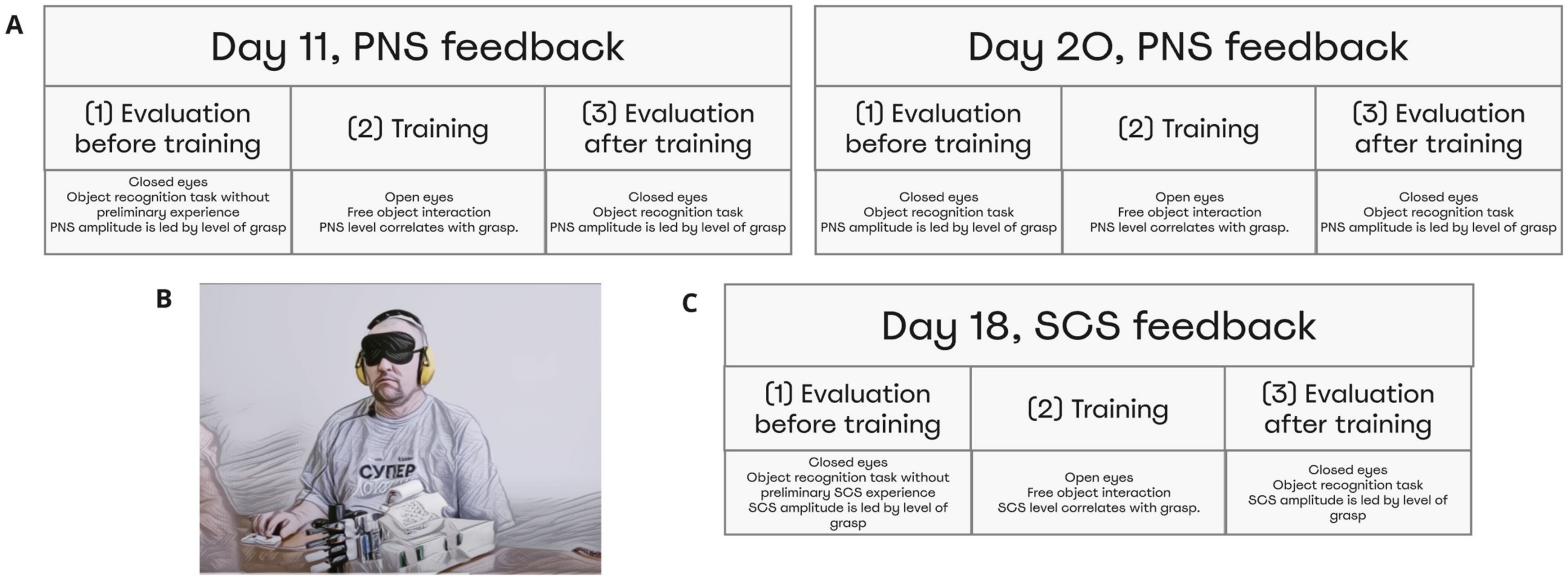
Object size detection experimental design and chronology. Object size detection was conducted thrice on day 11, day 20, and day 18. Each time experiment was held in three stages: Evaluation before training, Training, and Evaluation after training. Before training and after training subjects differentiated objects of different sizes using neurostimulation feedback that caused a sense of phantom hand grasping. **(A)** During day 11 and day 20, patient S12 and S13 completed the task using PNS. **(B)** Depicts the full setting that included a blindfold and sound cancellation headphones. **(C)** During day 19, patient S12 completed the task using feedback from SCS.

The object-grasping sessions aided by PNS were conducted on postsurgery days 11 and 20 (Figure 3A). Additionally, SCS was used in patient S12 during the object-grasping session conducted on day 19 (Figure 3C). Each session consisted of three parts: (1) evaluation before training, (2) training, and (3) evaluation after training. The patients were instructed that an object would be grasped by the prosthetic hand and his task would be to determine whether the object size was small, medium or large. Each trial started with PNS amplitude being lower than the sensory threshold. The cylinders were placed in front of the subject manually by the experimenter, which took about 3 s.

During the evaluation sessions (parts 1 and 3 of the session), subjects wore a mask and noise-isolating headphones (3 m Peltor; Figure 3B). During the evaluation that preceded the training (part 1), the subjects did not have any prior knowledge in this task. Yet, they experienced a range of evoked sensations, which provided an opportunity of testing whether the subjects could associate the level of PNS magnitude with the prosthetic aperture size. During the training period (part 2 of the session), participants had a full vision of the prosthetic hand and the cylinders. They were allowed to freely interact with the objects.

#### 2.3.2 Statistical analysis

For each session, accuracy metrics and confusion matrices of object size prediction were computed. Accuracy was measured as the proportion of trials where the object was identified correctly. Permutation tests were used to assess statistical significance of prediction accuracy. Namely, the subjects’ answers were randomly shuffled 700 times to compute a statistical distribution of random accuracy. Next, the real experimental accuracy was compared with the permutation distribution to obtain the *p*-value. We used the Wilcoxon test (Python, Scipy) to compare the mean accuracy from the random permutations with the actual value of accuracy observed for the object size recognition session.

### 2.4 Softness detection

#### 2.4.1 Experimental design

**Figure 4:**
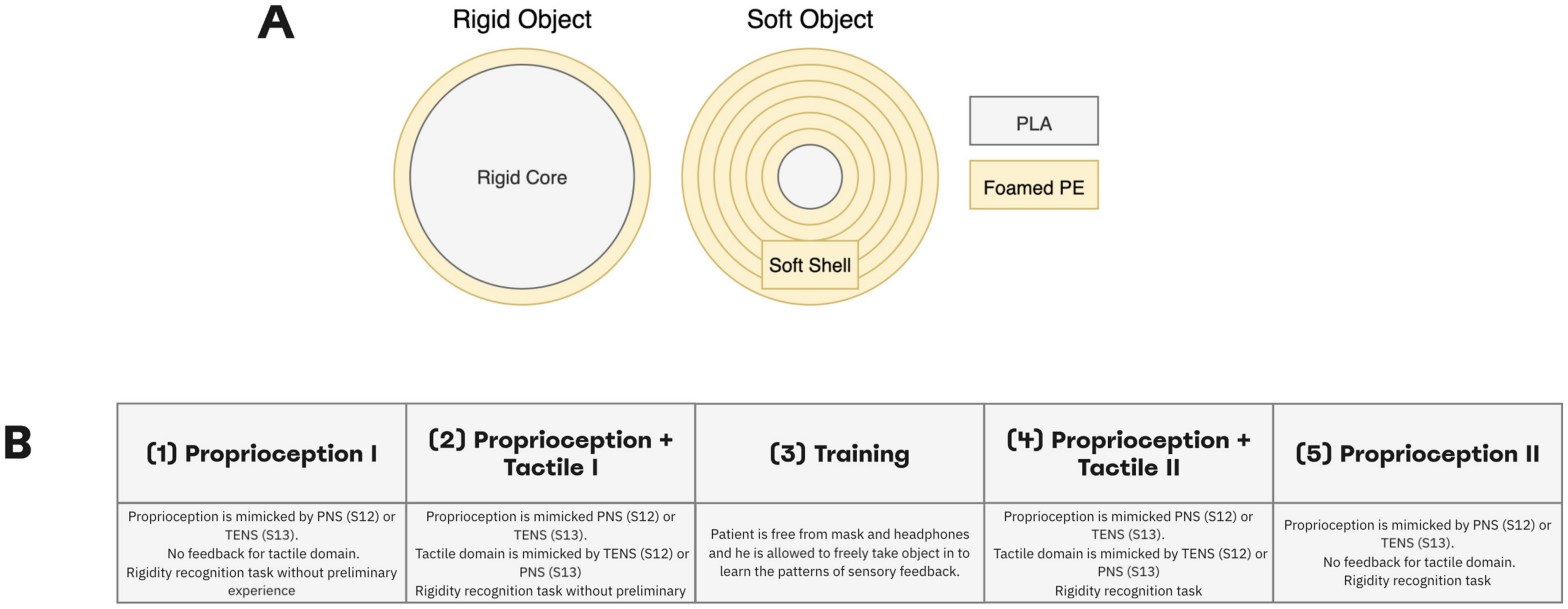
Softness detection experimental design. (A) Participants needed to differentiate between rigid and soft objects. The soft object was assembled by wrapping a 20-mm rigid core with a soft foamed polyethylene of 2-mm thickness. For the rigid object, the inner core diameter made up about 58-mm. Both objects in non-compressed state were approximately 60 mm in outer diameter. (B) Experiment was conducted in four stages: Proprioception I, Proprioception plus Tactile I, Proprioception plus Tactile II, Proprioception II. During the experiment, the subjects differentiated object rigidity using PNS (S12) or TENS (S13) stimulation only in sessions of Proprioception I and Proprioception. In sessions of Proprioception plus Tactile I and Proprioception plus Tactile II using PNS and TENS stimulation simultaneously.

In the softness detection test, we determined how well patients could use a combination of PNS and TENS to discriminate objects of different softness (Figure 4A). Both objects in non-compressed state were approximately 60 mm in the outer diameter (Video 2). The neurostimulation was constructed to mimic two somatosensory modalities. In each subject, a pair of electrodes was placed on the shoulder to produce TENS (50 Hz, 50 μs). For S13, the TENS component mimicked proprioception as it provided information of the prosthetic-hand aperture. The PNS component mimicked tactile sensations from the fingertips, and the appropriate signals were derived from the pressure sensors of the prosthetic fingers. For S12 we used an opposite arrangement: PNS mimicked proprioception and TENS mimicked tactile sensations. This change was done because patient S12 associated PNS with proprioception but patient S13 associated it with tactile sensations.

The sequence of task events was similar to the object size detection task. An experimenter placed an object in front of the prosthetic hand in a randomized order and then gently taped the patient’s arm to signal the trial start.

The experimental session consisted of four sessions of object softness recognition with a training procedure in between (Figure 4B). We call the first part “Proprioception I” because only the proprioceptive mode was turned on, and a subject needed to differentiate objects using only PNS (for S12) or TENS (for S13) feedback. In the second part called “Proprioception plus Tactile I” both types of stimulation were turned on allowing assessment of object softness. A patient was wearing a mask and soundproof headphones. Prior to these sessions, the subject did not have any experience in this task.

Next, the subject was free of headphones and mask during the training session. He was allowed to freely manipulate objects while the combination of TENS and PNS enabled the sensations. Then, the patient wore the mask and headphones and completed the session called “Proprioception plus Tactile II” where both TENS and PNS amplitude modulation guided the subject during object softness recognition. Finally, during the part “Proprioception II” session, one of the simulation channels was turned off while the remaining one (PNS in S12 and TENS in S13) mimicked proprioception. Forty trials per session were run.

#### 2.4.2 Statistical analysis

We estimated accuracy and confusion matrices, the same way as it was done in the analysis of object size detection. Then, permutation tests were used to assess statistical significance. Then, the Wilcoxon test (Scipy, Python) was applied to compare mean accuracy from the random permutations with the actual accuracy observed for the session of object size recognition.

### 2.5 Stimulation settings for sensory discrimination tests

For each test of the object size recognition, stimulation parameters were selected based on the preceding sensory mapping. An electrode pair was used that mimicked proprioceptive sensation in the phantom hand. The range of stimulation amplitudes was chosen to be comfortable for the subject and corresponded to the psychometric-scale values from 1–2 (barely perceivable) to 7–8 (massive yet comfortable perception). The readings from the prosthetic finger encoders provided a measure of the prosthetic-hand aperture, which changed from 0% (fully closed) to 100% (fully opened). This signal was converted into the amplitude of PNS or SCS.

**Figure 5:**
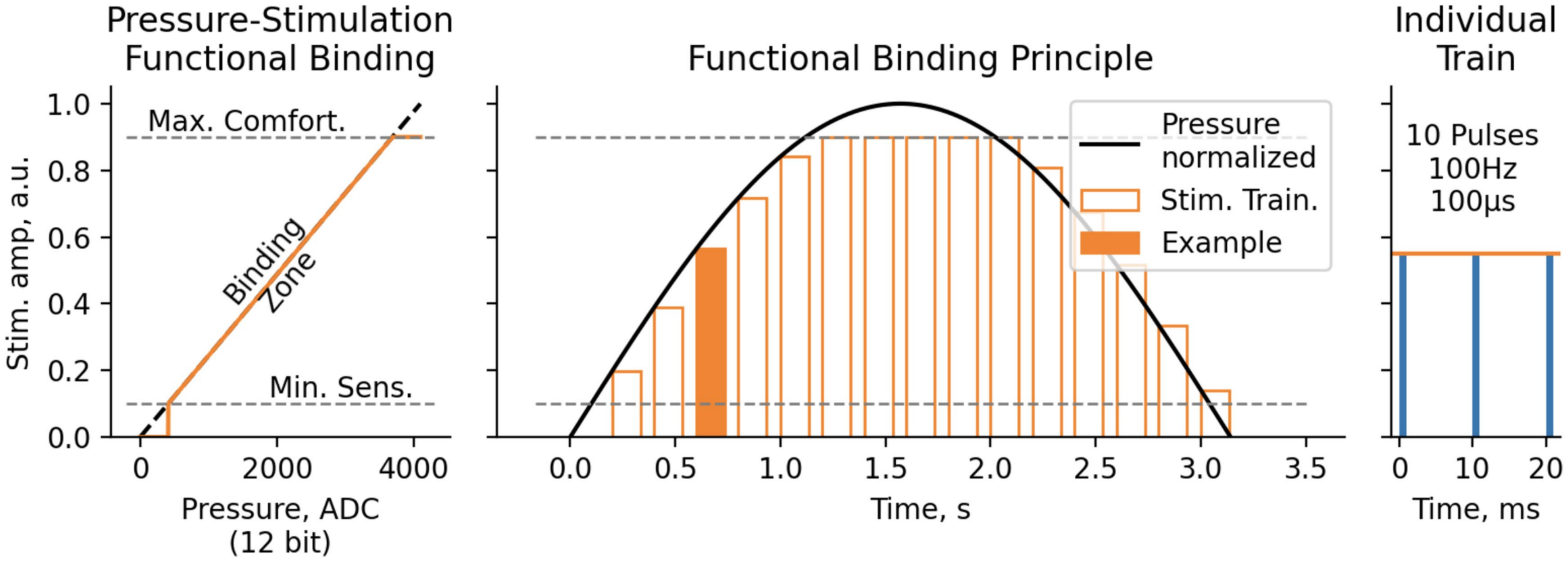
Functional binding principle for Softness and Object size detection test. Pressure values, measured with 12-bit ADC ([0.0.4095] range), were then translated to electrostimulation amplitude linearly between Minimal Sensitive Stimulation threshold (1–2 Pts) and Maximal Comfortable Stimulation threshold (7–8 Pts). The stimulation was performed in trains of 10 pulses 100 μs each, with pulse rate of 100 Hz and train rate 4.6 Hz.

In the softness detection experiment, information from the bionic finger encoder was complemented with the signals from the pressure sensors (Optical Tensometers by Motorica LLC) placed on the prosthetic fingertips. The sensor readings were scaled, so that 0% corresponded to a fully open prosthetic hand and 100% corresponded to the highest pressure during the grasping. The signals from the pressure sensors were converted into TENS (for S12) or PNS (for S13) amplitude with a linear transfer function. Stimulation amplitude did not exceed the psychometric values of 7–8 (Figure 5). The corresponding processing was done using a laptop computer and NimEclipse simulator. The aperture was sampled at 30 Hz and pressure readings were sampled at 100 Hz whereas the PNS and TENS amplitudes were updated at 10–30 Hz depending on the stimulator parameter update latency. PNS and TENS trains were delivered at the frequency of 4.6 Hz. The stimulation trains were arranged as 10 pulses with a width of 100 μs presented at 100 Hz.

### 2.6 EEG recordings

#### 2.6.1 Experimental design

EEG recordings were conducted using the standard 10–20 montage system with 32 channels. An NVX-136 amplifier (Medical Computer Systems LLC) was used. The ground electrode was located on the forehead. The mean of A1 and A2 channels was subtracted as a reference.

A patient comfortably seated while neurostimulation was delivered. Electrical stimuli with constant frequency (100 Hz) and pulse width (900mcs) were provided using peripheral nerve stimulation (PNS) and spinal cord stimulation (SCS). Stimulation amplitude was selected individually according to the participant’s reported sensations. Sensations at the level 5–7 of the psychometric scale were chosen. Two sessions of EEG recordings were conducted where the stimuli had different durations: long and short. For patient S13, the long and short stimuli had the durations of 1 s and 0.5 s, respectively, and for patient S12 the durations were 1 s and 0.3 s. The selection of stimulus duration was constrained by the technical limitations of the neurostimulator. During each session, the stimulus was presented 100 times with a variable interstimulus interval (3.6–4.4 s).

#### 2.6.2 EEG analysis

EEG data were analyzed using MNE Python (Gramfort et al., 2013). Data was notch filtered at 50 Hz and bandpass in the interval 1-40 Hz. Next, an ICA was applied, with the use of the ALICE toolbox (Soghoyan et al., 2021) to remove ocular artifacts. Noisy channels were dropped from the recording. Additionally, noisy epochs were removed based on a 150-μV threshold. All epochs were split into four condition, according to the type and duration of stimulation: PNS_long, PNS_short, SCS_long and SCS_short. Evoked response potentials (ERPs) were computed for each condition by averaging among remained epochs. We estimated the components P1 and N1 as average values within a 30-ms window around the ERP positive and negative peaks, respectively.

#### 2.6.3 Statistical analysis

For the condition PNS_long and SCS_long, we calculated the value of lateralization by comparing the average activity in two regions of interest (ROI) located in the right and left somatosensory cortices: C4, CP2 and CP6 versus C3, CP1, and CP5. These are the areas where we expect evoked sensation to be processed. The amplitudes of components N1 and P1 were calculated for each ROI and for PNS and SCS separately.

Changes in the ERP components were assessed over the course of the experimental session. The data for conditions PNS_long and SCS_long was split into two halves. Next, we compared the first half to the second to obtain a measure of response change over time. All statistical comparisons were conducted using paired t-tests (Python, SciPy) and adjusted using FDR correction for multiple comparisons.

### 2.7 Embodiment

To test whether neurostimulation-based feedback had an effect on the prosthetic hand embodiment, the patients were asked to fill the appropriate questionnaire (Marasco et al., 2021). The questions were translated to their native Russian language. The questionnaire was completed four times:

1. on day 9 (baseline estimation), after the patients used the prosthetic hand for the first time without sensory feedback in both patients,
2. on day 11, after the first session of object size-detection in both patients,
3. on day 20, that is after the second session of object size-detection in S12, and after the second session of object size-detection and the session of softness detection in S13, and.
4. on day 25, after the session of softness detection in S12 only.

The survey required the participants to express their degree of agreement with 10 statements: three statements of predicted phenomena, six control statements and the last statement we added to estimate the state of phantom limb pain during the active tasks (“I felt that during the work with prosthesis my phantom limb pain decreased”). The agreement to the statements ranged from −3 (totally disagree) to 3 (totally agree).

## 3 Results

### 3.1 PNS and SCS naturalness assessed with sensory mapping

**Figure 6:**
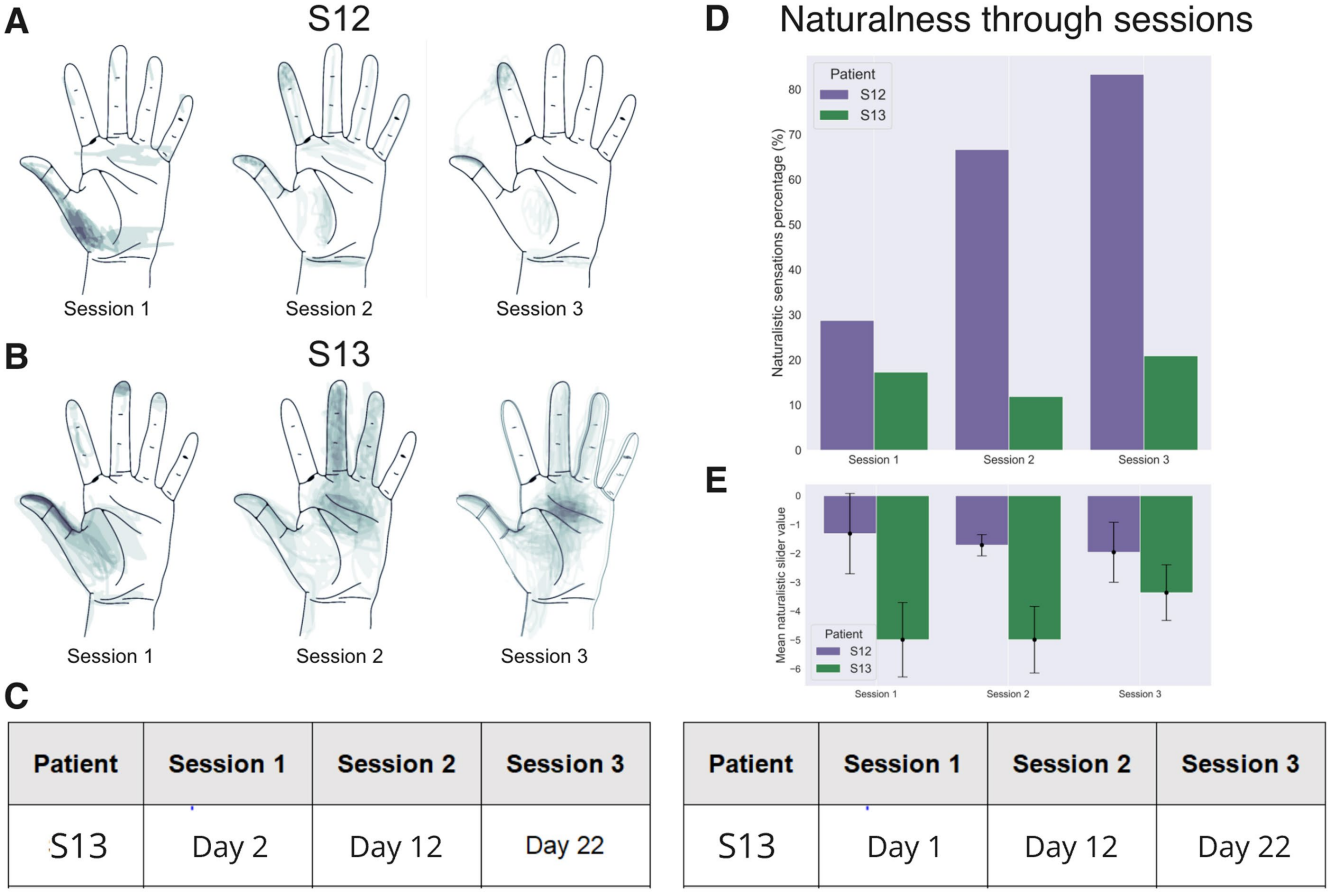
Sensory mapping results. Data for patients S12 and S13 collected in three mapping sessions are shown, conducted with an interval of ~10 days. (A,B) Drawings of stimulation projection zones in patient S12 and S13, respectively. Shading corresponds to the density of reported sensation locations. In both patients, the evoked sensations initially concentrated around the thumb but shifted toward the other fingers during the 2nd and the 3rd mapping sessions. (C) A table showing the dates when the mapping sessions were conducted. Day 1 is the next day after the implantation surgery. (D) Percentage of naturalistic and non-naturalistic sensations by the mapping session. This percentage markedly increased in patient S12 but stayed approximately the same in patient S13. (E) The average value of the analog naturalness slider across sessions. Positive values denote a sensation that was experienced as a natural and familiar to a subject. While, negative values represent odd somatic sensations that the subjects have not experienced during their daily routines.

The sensory mapping of the PNS was conducted several times throughout the study (Figure 6C). For some electrode pairs the location of evoked sensations on the phantom hand was constant for as long as 24 days following the implantation surgery. In patient S12, stimulation with each electrode pair evoked sensations in the thumb. Additionally, in 18% of cases, sensations appeared in the other fingers (Figure 6A). A migration of the thumb sensations occurred throughout the days. By experimental day 12, they shifted from the base of the thumb to its fingertip and the index finger fingertip. Concurrent with this shift, the patient started to report proprioception-like sensations of the thumb and index finger being flexed during PNS. Additionally, the patient reported naturalistic sensations more frequently. By the third session (day 22), the rate of naturalistic descriptions increased to 84% from the initial 29%.

In patient S13, several parameters of stimulation resulted in the sensations of touch, pressure and itch (Figure 6D). A significant increase in sensation naturalness occurred by the last mapping session (Figure 6E) as compared with the first (Mean diff. = −1.450; *p*-adj = 1.082e-7; Tukey HSD) and the second (Mean diff. = −1.629; *p*-adj = 4.309e-10; Tukey HSD) session. Similarly to patient S12, it is possible to evoke sensations in the tip of the thumb and the middle of the palm with a pair of electrode sites (Figure 6B).

In summary, measurements of several parameters showed that the degree of naturalness of the evoked sensations increased with practice in both subjects. Notably, in both participants, we observed a migration of the projection zones from the palm to the fingertips during sessions 2 and 3. This result could be related to neuroplasticity induced by the continued use of neurostimulation including the object-size discrimination tasks.

### 3.2 Spinal cord stimulation

**Figure 7:**
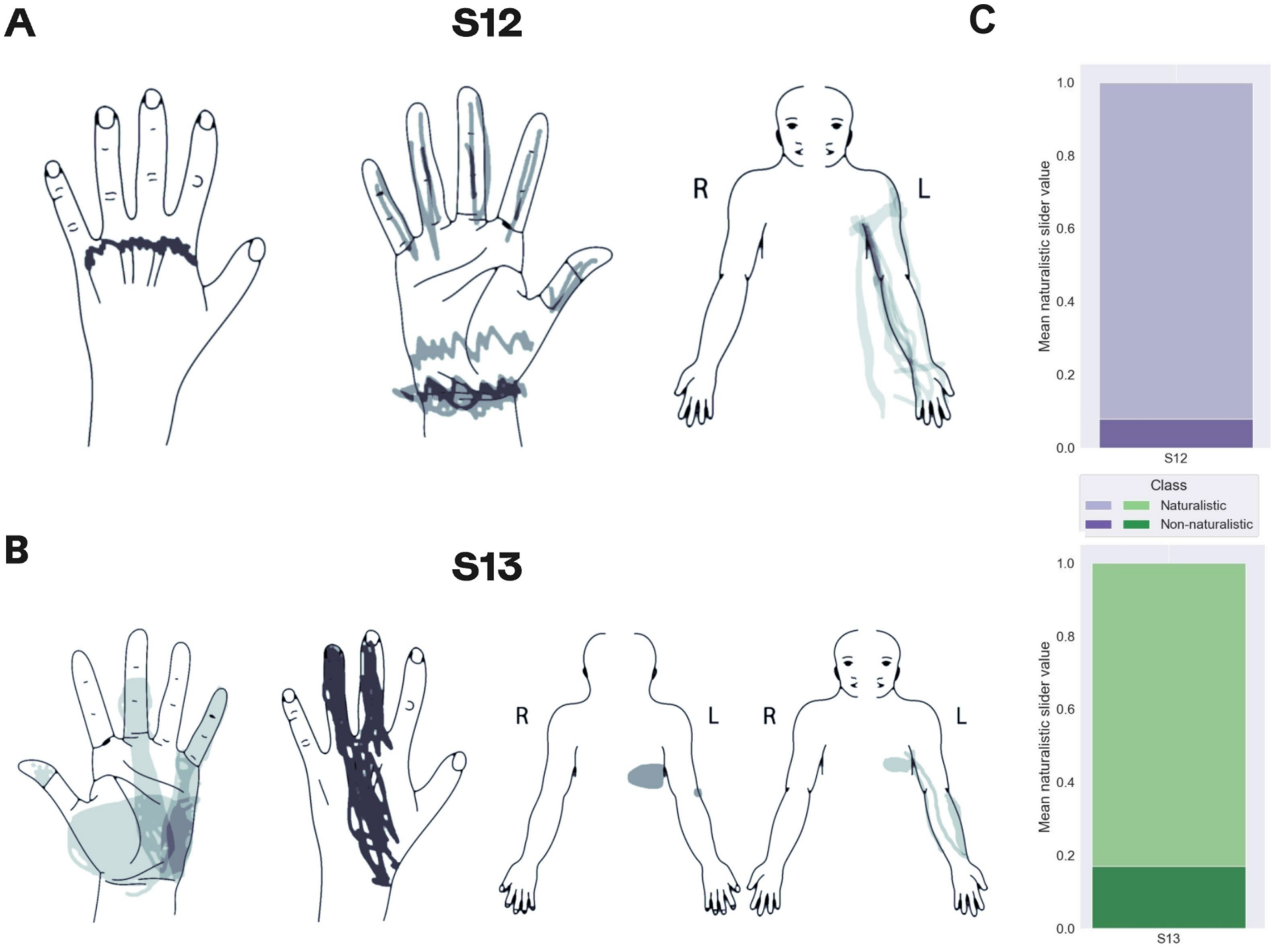
Mapping of sensations evoked by spinal cord stimulation. (A,B) Maps for patients S12 and S13, respectively. Shading density corresponds to the frequency of reported locations. (C) The proportions of the reported naturalistic and non-naturalistic sensations.

The comparison of PNS and SCS in terms of naturalness and localization of projections showed that in both patients SCS projected to the phantom hand, but also to the arm and trunk. Patient S12 experienced sensations in the phantom palm in 20% of SCS trials while for PNS each trial was associated with the perceptions in the thumb or pointing finger (Figure 7A). In patient S13, SCS sensations in the phantom hand were evoked for 36% of the tested electrode configurations, and PNS projected to the phantom hand for 68.8% of the cases (Figure 7B).

With respect to sensation naturalness, SCS tended to generate less naturalistic sensations compared to PNS. Only 8 and 17% of the sensations evoked by SCS were marked as natural by patients S12 and S13, respectively (Figure 7C). Patient S12 rarely reported sensations of hand movements during SCS whereas such sensations were often induced by PNS. In patient S13, SCS and PNS evoked similar sensations.

### 3.3 Object size detection improves by training day

**Figure 8:**
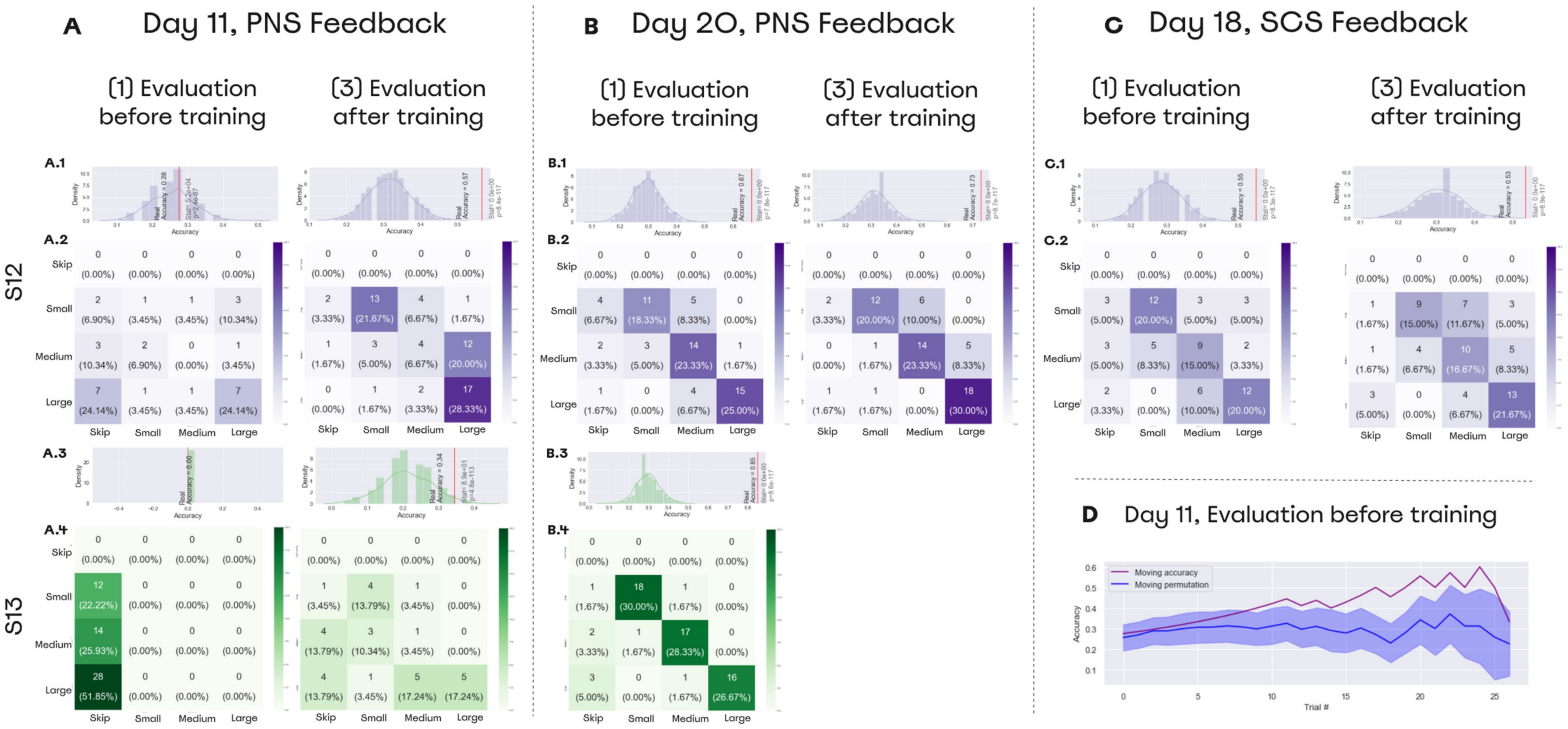
Performance on object size discrimination. (A–C) Confusion matrices for object size discrimination. The histograms shown on top of the confusion matrices represent distributions for random accuracy obtained with permutations. Red line represents the real accuracy obtained from the experimental data, and Wilcoxon test statistics are shown. (A) Day 11 data. On that day, both subjects improved their performance following a training session. (B) Day 20 data. Both subjects completed the task with the accuracy exceeding 65% before training was conducted. (D) Day 19 data. Patient S12 discriminated object size with accuracy over 55% using SCS-based feedback. (D) Performance data for day 11. During “Evaluation before training,” patient S12 correctly discriminated object size without any prior training. The floating mean for the accuracy (purple line) is plotted together with the random-performance values computed using permutations (blue line). With 700 permutations, distribution was assessed for performance accuracy on each set of trials. Floating random accuracy is plotted, with transparent shadow representing standard error.

Both subjects reported the size of an object grasped by the prosthetic hand (Video 1) with an accuracy exceeding the chance level of 33% (Figure 8). On day 11, patient S12 improved the accuracy from 28 to 57%. During the “Evaluation after training” session, the patient erroneously marked medium objects as large ones. On day 20, the patient’s accuracy reached 67% which was significantly higher than random performance computed using permutations (the entire permutations statistics are presented in Figure 8). Notably, accuracy for the medium-size objects increased from 6.67 to 23.33%. The Day-20 final score following the training was 73.33% (Figure 8B). Before the training S13 skipped all the trials, but following the training, accuracy reached 34%. On day 20, S13’s accuracy increased to 85% before additional training was conducted. In the control session without stimulation, both subjects were unable to discriminate object size.

During the “Evaluation before training” session, subjects associated the neurostimulation-evoked sensations to the object size without any previous training. Patient S13 could not discriminate object size without training and skipped all the trials of the evaluation session. In contrast, S12 started making correct guesses. Starting from the 10th trial, accuracy exceeded random performance (Figure 8D).

In the SCS sessions, patient S12 recognized objects before training with an accuracy of 55% and after the training with an accuracy of 53% (Figure 8C).

### 3.4 Softness detection with a combination of PNS and TENS

**Figure 9:**
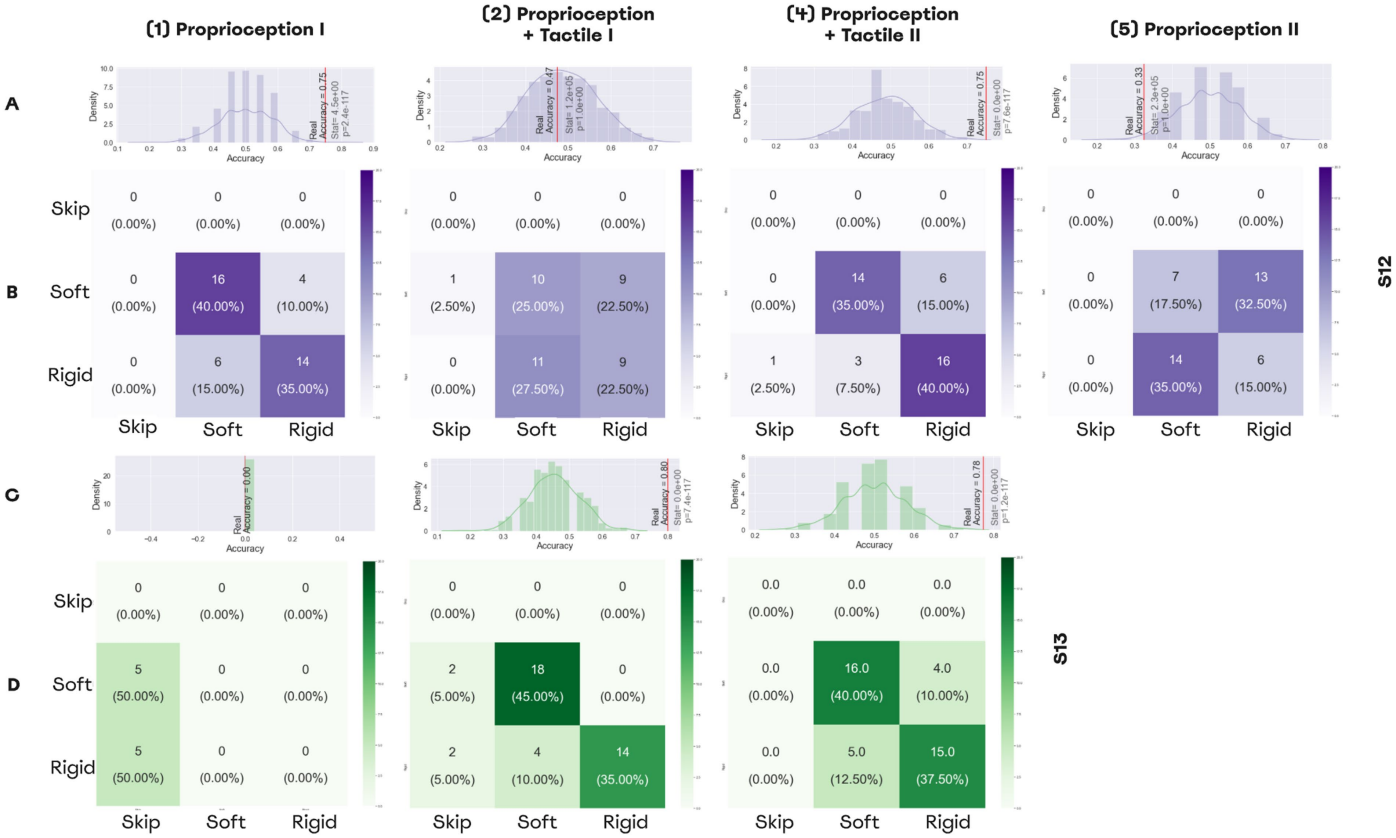
Performance in softness detection task. (A,C) Histograms representing the distributions for random performance accuracy calculated using permutations. The red line indicates accuracy for the actual experimental data. Wilcoxon test results are shown, as well. (A) The distribution for the permutations obtained from the answers of patient S12, (C) The distribution for the permutations for the answers of patient S13. (B,D) The confusion matrices for the sessions of softness detection, (B) The confusion matrices for patient S12, (D) The confusion matrices for patient S12.

Both subjects learned to perform the softness detection task (Figure 9, Video 2). Using only proprioceptive feedback, patient S12 could differentiate objects with an accuracy of 75% before the training. After the addition of the second sensory channel, accuracy decreased to 47.5% (Figure 9A). After the training accuracy reached 75% (Figures 9A,B), but when the task switched back to the proprioception-only mode, accuracy decreased to 32.5%.

For patient S13, the first session was shorter due to technical reasons, and during all trials he was skipping answers without making guesses. By contrast, with an additional PNS feedback that represented the tactile modality, the patient discriminated object size with an accuracy of 80%. Following the training, accuracy was 77.7% for the dual-sensory paradigm (Figures 9C,D).

### 3.5 Embodiment

**Figure 10:**
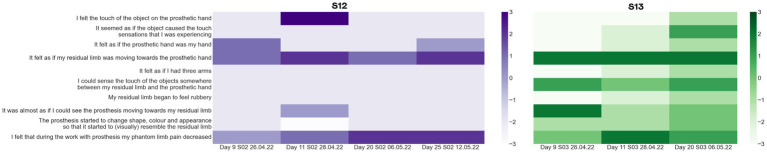
Prosthetic embodiment estimation. S12 and S13 were asked to fill the embodiment questionnaire which estimated if neurostimulation-based feedback had an effect on the prosthetic hand embodiment. These measurements were taken after each test of size and rigidity detection. The baselines estimation of embodiment was measured on day 11. The color of the matrix represents the level of agreement with the statement from −3 (Totally disagree) to 3 (Totally agree).

In patient S12, the embodiment increased from the baseline estimation of −3 to −1 by day 11, and then decreased to −6 by day 25. The statement with the highest variability in the answers was “I felt as if my residual limb was moving toward the prosthetic hand.” The score for this statement increased during the first session of neurostimulation-based feedback but decreased back after the second one. For the three questions from the control group (Figure 10), the answers changed dramatically. In patient S13 embodiment increased from −9 for the baseline estimation to −7 and to −1 for the first and second sessions, respectively. Similarly, he changed the answers to five out of six control questions. Notably, both subjects “Agreed” or “Totally Agreed” with the statement that PLP was suppressed during the tests of object size and softness discrimination.

### 3.6 ERP lateralization and adaptation to SCS and PNS

**Figure 11:**
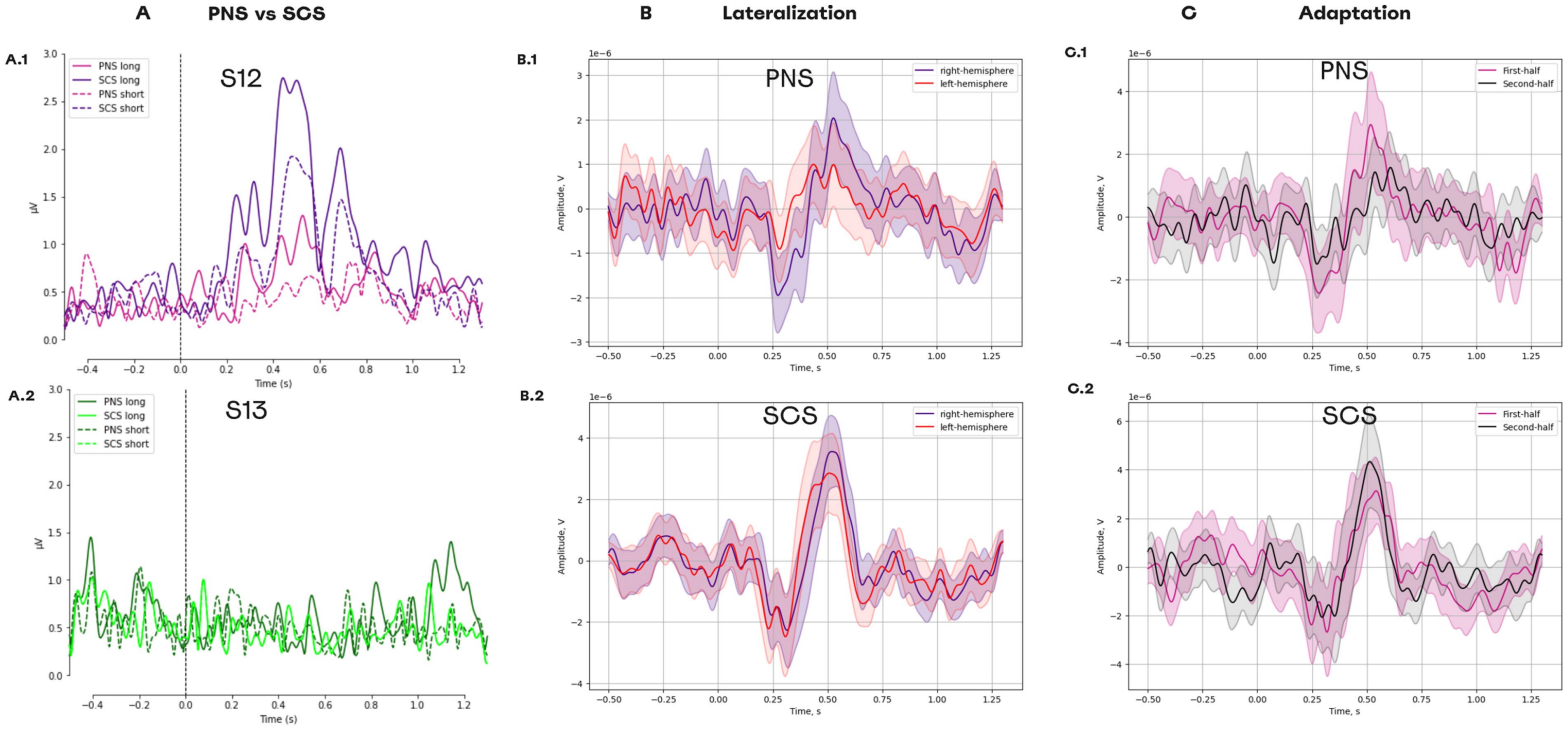
Evoked response potentials for different types of neurostimulation. (A) ERPs for the following conditions: PNS_long, PNS_short, SCS_long, and SCS_short. (A1) Data for patient S12. (A2) Data for patient S13. (B) ERPs in different hemispheres. (B1) The responses to PNS. (B2) The responses to SCS. (C) Change in ERP components over the course of the experimental session. (C1) The responses to PNS splitted into two halves. (C2) The responses to PNS splitted into two halves.

In patient S12, stimulation resulted in a clear evoked response potential (ERP). The ERP was stronger for SCS than for PNS (Figure 11A) and was stronger for the longer stimulation. In patient S13, no clear ERPs were found. The comparison of long SCS to long PNS revealed a stronger lateralization during PNS for the component N1 [t(98) = −4.605, *p* = 0.000, paired *t*-test] and P1 [t(99) = 2.892, *p* = 0.017, paired *t*-test; Figure 11B]. For SCS, lateralization was weaker for N1 [t(98) = −0.073, *p* = 0.942, paired *t*-test] and P1 [t(98) = 2.797, *p* = 0.017, paired *t*-test].

For PNS, a reduction was found for the components P1 [t(49) = 2.265, *p* = 0.056, paired *t*-test] which had a higher amplitude for the first half of the trials than for the second half (Figure 11C). Such adaptation was not detected for SCS condition, since no reduction occurred in P1 [t(48) = −1.374, *p* = 0.234, paired *t*-test].

### 3.7 Suppression of phantom limb pain

**Figure 12:**
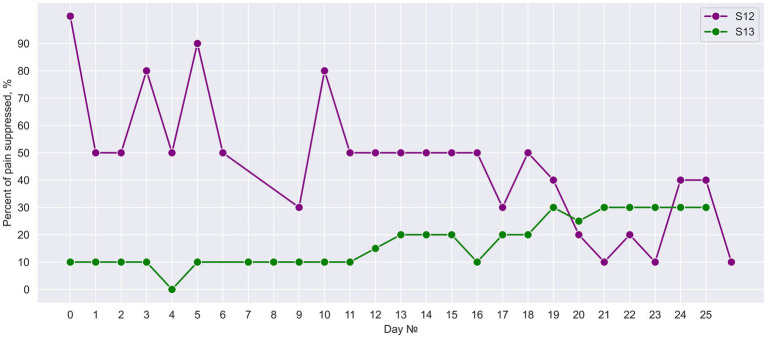
Daily suppression of phantom limb pain. The percent of PLP suppression during 25 days of neurostimulation treatment.

Neurostimulation resulted in PLP suppression in both subjects with the effect average value of 46.00% ± 22.98% (Mean ± St. dev) and 17.20% ± 8.84% in S12 and S13, respectively. Both subjects reported that pain intensity swung during the day without any consistent pattern. In patient S13, PLP further decreased after PNS and SCS were combined on day 13 (Figure 12). Before this combination was made, PLP suppression was 9.58% on average, and after day 13 it was 24.58% on average.

## 4 Discussion

In this study, PNS and SCS evoked somatosensory sensations in the phantom palm of two upper-limb amputees. The naturalness of sensation improved as the patients practiced with neurostimulation, and it tended to be higher for PNS than for SCS. EEG recordings showed that PNS resulted in more lateralized responses as compared to SCS, with a faster attenuation of experienced sensations. The neurostimulation-based prosthetic feedback enabled both patients to detect the size of an object grasped with a bionic hand. Detection accuracy improved as the patients practiced on that task. Moreover, by combining PNS and TENS to recreate the tactile and proprioceptive sensations simultaneously, sensing of object softness was enabled.

### 4.1 PNS and SCS for neuroprosthetic sensations

Somatosensory substitution systems were previously developed for the amputees using haptics devices attached to the residual limbs (Muijzer-Witteveen et al., 2016). Alternatively, PNS-based systems could evoke sensations in the phantom hand which, during grasping performed with a bionic hand, represent characteristics of an object being grasped (Soghoyan et al., 2023). In the current study, we showed that sensory discrimination accuracy in this task improves and reaches 85% after a prolonged practice. Yet, the sensations induced by PNS are intuitive enough even to a naive user. One of the participants (S12) detected the size of three objects without any prior training which seemed to rely on mechanisms of perceptual learning (Bedford, 1993). To our knowledge, this is the first demonstration of a PNS-based prosthetic not requiring any prior perceptual training on sensory discrimination tasks. We suggest this is the strongest argument for the possibility of the restoration of naturalistic sensation to the amputees. Such sensory-restoration systems are much needed by the upper-limb amputees to operate their prostheses efficiently (Kyberd et al., 2007). Moreover, during the sensory discrimination tasks, all patients reported that their PLP was suppressed, which makes our sensory restoration approach applicable for both sensory substitution and PLP treatment.

SCS is the other invasive-neurostimulation approach to somatosensory feedback (Nanivadekar et al., 2022; Chandrasekaran et al., 2020). We found that with SCS used as sensory feedback from a bionic hand, one patient (S12) could discriminate the size of the objects being grasped. This demonstration adds to the previous literature on the other users of SCS and illustrates that different types of neurostimulation can be used for prosthetic sensations in the same participant. Moreover, we showed that the experience in sensory discrimination aided by PNS could be transferred to the same task aided by SCS.

SCS evoked less focal sensations compared to PNS. Additionally, changing the electrode pair altered the evoked sensation significantly. With respect to utilizing SCS for neuroprosthetic sensations, this finding means that generating tactile sensations in small areas of the phantom hand is more difficult with this method compared to PNS. Yet, some improvements in prosthetic sensations could be achieved by adjusting the pulse amplitude and width, and by creating complex electrical fields with several electrodes.

### 4.2 Dual neurostimulation for somatosensory integration

The direct integration of proprioceptive and tactile submodalities of somatosensation is a crucial part of our daily motor behavior. The most that is known about the mechanisms of somatosensory incorporations has been collected in nonhuman primates’ studies (Iwamura, 1998; Schieber, 2001; Delhaye et al., 2018). Lesion of the critical areas (such as Brodmann area 2) results in inability to perform motor coordination tasks (Delhaye et al., 2018). Thus, the development of neuroprosthetics systems that restore both modalities is key. For example, identification of an object’s softness requires integration of tactile information with the information about the configuration of the hand. Previously, such tasks with amputees were completed only with the systems assembled with the use of TMR and vibromotors (Marasco et al., 2021). We developed the first system that incorporates PNS and TENS for this task.

In patient S12, PNS was employed to mimic proprioception because it caused mostly kinesthetic sensations when applied alone. By contrast, patient S13 experienced tactile sensations when PNS was applied, so PNS was chosen to mimic tactile sensations when combined with TENS, and TENS mimicked proprioception. Patient S13 successfully used these two sensory streams to differentiate between the soft and rigid objects with an accuracy of 80%. Notably, he was unable to complete this task using only TENS that mimicked proprioception.

### 4.3 Naturalness of neuroprosthetic sensations

The naturalness of evoked somatosensory prosthetic sensations is ussually assessed with questionnaires (Cuberovic et al., 2019; Chandrasekaran et al., 2020; Heming et al., 2010). To achieve a higher naturalness, in some studies the stimulation current was modeled to mimic the normal sensory nerve action potential (SNAP; Okorokova et al., 2018). Such biomimetic approaches provide a more natural feedback and allow a higher performance in motor tasks with a prosthesis (George et al., 2019). The other approach to enhance naturalness using PNS stimulation is the development of specific electrode configurations that enact focal stimulation of distinct nerve fibers (Clark et al., 2014; Yildiz et al., 2020; Charkhkar et al., 2019).

A case study (Cuberovic et al., 2019) reported that the sensation of naturalness increased during a daily use of a PNS-based prosthesis during the first month of use. We observed a similar trend of growing naturalness for the sensory mapping sessions for S12 and S13. This pattern of increasing naturalness matches the adaptation to cochlear implants (Carlson, 2020). Patients with cochlear implants experience mechanical and high-pitched sounds during the first 3–6 months of use. In the first 6 months rehabilitation implies active adaptation to the new way of sound with an additional adjustment after. Such changes in natural perception may be explained by the mechanisms of sensory normalization and sensory adaptation which exist in the somatosensory domain too (Brouwer et al., 2015). Some short-term adaptation to PNS stimulation was indicated in this study using EEG ERP, but additional long-term changes are in high interest for the following studies.

Since our patients were implanted with both PNS and SCS electrodes, we are the first to have examined the difference in sensations induced by these types of neurostimulation in the same participants. Patients reported the sensations felt as more natural during the PNS sensory mapping, especially S12. The main limitation for a more general conclusion is that this could have happened because medical specialists applied PNS as the main neuromodulation technique for his PLP suppression which wasn’t the case for S13.

To summarize, we have shown that sensation naturalness can be achieved by: (1) appropriate spatial placement of the electrodes, (2) biomimetic current characteristics and (3) rehabilitation that induces sensory adaptation.

### 4.4 Embodiment

One of the key benefits of PNS and SCS based feedback in neuroprosthetics is the increased sense of embodiment that was reported in many case studies (Raspopovic et al., 2021; Rognini et al., 2019). In our research, we estimated it using the questionnaire (Marasco et al., 2021), which is a common approach for estimation of prosthesis embodiment. It was originally derived from the rubber hand illusion (RHI) questionnaire, and it contains a set of targets and control questions. Somewhat unexpectedly, both subjects responded variatively to the set of control questions, which makes the result of the questionnaire controversial. Recently, the problems of embodiment definition were highlighted in a respective review (Zbinden et al., 2022).

This result could be explained by an inexperience in prosthesis usage in our participants at the time the first assessment of embodiment was performed. None of the participants used the prosthesis prior to this study, which decreased the accuracy of the baseline embodiment estimation. Additionally, the control questions of RHI questionnaire have been previously criticized, especially for studies with amputees (Riemer et al., 2019). These issues could be avoided by using implicit ways of embodiment estimation, such as the ones based on sensory attenuation and cross-modal congruency (Zbinden et al., 2022). Additional insights on the embodiment could be provided by asking patients if their PLP changes when operating prosthesis with sensory feedback. After we added this question to the embodiment questionnaire, we found that PLP decreased in both patients during the active use of the sensorized prosthesis. We have discussed previously the importance for PLP suppression of an active use of prosthetic sensations (Soghoyan et al., 2023).

### 4.5 EEG for objective estimation of evoked sensation

The direct stimulation of peripheral nerves is a well-known method for the investigation of aspects of somatosensory processing using ERP (Muzyka and Estephan, 2019; Aminoff, 1987). We employed this method for the comparison of effects of PNS and SCS. PNS caused a more lateralized response in comparison with SCS. This effect is in high agreement with the behavioral data of lateralized responses that were collected during PNS sensory mapping. The reported decrease in the P1 component during the recording for PNS stimulation seems to be the result of somatosensory adaptation (McLaughlin and Kelly, 1993). Here we did not collect data about the stimulation-induced perceptions during the EEG recordings to compare the perceived intensity with EEG response amplitude, but such correlation was shown in previous studies (Johnson et al., 1975). These electrophysiological markers of stimulation could objectify sensory mapping procedures. Also, the objective representation of stimulation could be used for a closed-loop neurostimulation that will adjust stimulation amplitude to cause the required level of perceived sensation. Previously, we suggested using EEG biomarkers to adjust stimulation for the treatment of PLP (Kleeva et al., 2022).

### 4.6 Pain suppression

The use of PNS as a tool for PLP suppression is still in need of additional validation (Knotkova et al., 2021). We demonstrated the efficiency of such stimulation in our previous study (Soghoyan et al., 2023) and we added the case of S12 here. Remarkably, in S13 an additional decrease in PLP level was observed when simultaneous PNS and SCS were included in his treatment program. Classically neuromodulation for neuropathic pain is treated within one paradigm of stimulation. Thus, a combination of two types of neurostimulation could improve the treatment. Yet, given a small number of patients in this study, much more research will be needed in the future, particularly the studies with `placebo and control groups.

### 4.7 Limitations and suggestions for future research

This study has several limitations. Only two patients were examined with different approaches to PLP treatment. In patient S12, PNS was used to treat PLP. In patient S13, SCS was used for this purpose followed by using a combination of SCS and PNS. These types of neuromodulation delivered throughout the days could have affected the results obtained during the experimental sessions, including the perception of different stimuli, performance of sensory discrimination tasks, and changes in prosthetics embodiment. The test schedules were slightly different in patients S12 and S13 because of the availability of required equipment and the directions from the medical specialists.

Additionally, the small sample size makes it difficult to assess the changes in embodiment statistically. The embodiment was assessed explicitly using a questionnaire. This assessment was done three times in patient S12 and four times in patient S13, which is insufficient for running statistics. In the future, embodiment assessment could be improved by making more measurements throughout the course of training. Additionally, it would be beneficial to add implicit measurements of embodiment based on the phenomena of sensory attenuation and cross-modal congruency.

The future work with large samples of subjects should also include control groups. Moreover, when many patients are tested, they could be split into subgroups treated with PNS, SCS or their combinations. Extending the study duration to several months would be also beneficial, particularly for assessing patients’ improvement in object size and softness recognition tasks. It would be of interest for future research to test whether operating a sensorized prosthesis would be sufficient to suppress PLP without ongoing daily neurostimulation would be needed. The observation that PLP was reduced in patient S13 when PNS was combined with SCS hints that it would be valuable in the future to explore the other combinations of neurostimulation paradigms. Incorporation of cortical stimulation is of particular interest because it can both treat neuropathic pain (Moisset and Lefaucheur, 2019) and restore somatic sensations (Garcia-Larrea and Peyron, 2007). DBS of subcortical structures is the other potential approach which has already proved effective for managing essential tremor and Parkinson’s disease (Okun, 2012) and could alleviate neuropathic pain (Abreu et al., 2017). Notably, DBS of the thalamus is currently being researched as the method for enhancing visual (Panetsos et al., 2009) and somatosensory functions (Heming et al., 2010), so it would be of interest to test whether it could augment the effects of PNS or SCS. Overall, combining of several neurostimulation methods could improve the treatment of PLP.

Our EEG recording showed the difference in lateralization and adaptation in responses to PNS and SCS stimuli. These initial observations should be replicated in the larger samples of patients. Additionally, the effects of stimulation parameters on the EEG patterns should be studied in greater detail.

In the current study, a sensory mapping procedure was key for selecting the right stimulation parameters for evoking prosthetic sensations. This procedure was, however, time-consuming as the participants had to provide their subjective feedback via the questionnaires (Piliugin et al., 2024). Although lengthy, the sensory mapping provided valuable information, including the observation that the phantom-hand location where sensations were felt shifted over time. Cuberovic et al. (2019) reported similar shifts likely caused by neural plasticity. It should be noted, however, that Makin and Bensmaia (2017) argued that cortical maps of the body are not as plastic as suggested by the other literature, even in the amputees many years after limb loss. These conflicting results raise an important question: what drives plasticity in some neuroprosthetic systems but not in others?

Based on our present findings, we have more positive expectations compared to those of Makin and Bensmaia (2017) regarding the possibility of evoking of neural plasticity by the use of a neuroprosthesis. Yet, the exact mechanisms and brain regions involved remain to be elucidated and combining neurophysiological recordings with the participants’ reports should help to clarify this issue. Thus, our EEG recordings in patient S12 revealed an adaptation to median nerve stimulation as the patients’ sensations became more natural. Observations like that will help the future development of neural prostheses that blend with the brain representation of the body.

## Data Availability

The raw data supporting the conclusions of this article will be made available by the authors, without undue reservation.

## Footnotes

^1^ https://clinicaltrials.gov/
